# Toll-like receptor 2 promiscuity is responsible for the immunostimulatory activity of nucleic acid nanocarriers

**DOI:** 10.1016/j.jconrel.2016.12.029

**Published:** 2017-02-10

**Authors:** Malvina Pizzuto, Monique Gangloff, Daniel Scherman, Nicholas J. Gay, Virginie Escriou, Jean-Marie Ruysschaert, Caroline Lonez

**Affiliations:** a Structure and Function of Biological Membranes, Université Libre de Bruxelles, Boulevard du Triomphe, 1050 Brussels, Belgium; b Department of Biochemistry, University of Cambridge, 80 Tennis Court Road, Cambridge, UK; c CNRS, Unité de Technologies Chimiques et Biologiques pour la Santé (UTCBS), UMR 8258, F-75006 Paris, France; d INSERM, UTCBS U 1022, F-75006 Paris, France; e Université Paris Descartes, Sorbonne-Paris-Cité University, UTCBS, F-75006 Paris, France; f Chimie ParisTech, PSL Research University, UTCBS, F-75005 Paris, France; g Department of Veterinary Medicine, University of Cambridge, Madingley Rd, Cambridge CB3 0ES, UK

**Keywords:** Lipopolyamine (LPA), Lipoplex, Toll-like receptor (TLR), Nanocarrier, Docking, Gene therapy

## Abstract

Lipopolyamines (LPAs) are cationic lipids; they interact spontaneously with nucleic acids to form lipoplexes used for gene delivery. The main hurdle to using lipoplexes in gene therapy lies in their immunostimulatory properties, so far attributed to the nucleic acid cargo, while cationic lipids were considered as inert to the immune system. Here we demonstrate for the first time that di-C18 LPAs trigger pro-inflammatory responses through Toll-like receptor 2 (TLR2) activation, and this whether they are bound to nucleic acids or not. Molecular docking experiments suggest potential TLR2 binding modes reminiscent of bacterial lipopeptide sensing. The di-C18 LPAs share the ability of burying their lipid chains in the hydrophobic cavity of TLR2 and, in some cases, TLR1, at the vicinity of the dimerization interface; the cationic headgroups form multiple hydrogen bonds, thus crosslinking TLRs into functional complexes. Unravelling the molecular basis of TLR1 and TLR6-driven heterodimerization upon LPA binding underlines the highly collaborative and promiscuous ligand binding mechanism. The prevalence of non-specific main chain-mediated interactions demonstrates that potentially any saturated LPA currently used or proposed as transfection agent is likely to activate TLR2 during transfection. Hence our study emphasizes the urgent need to test the inflammatory properties of transfection agents and proposes the use of docking analysis as a preliminary screening tool for the synthesis of new non-immunostimulatory nanocarriers.

## Graphical abstract

**Figure:**
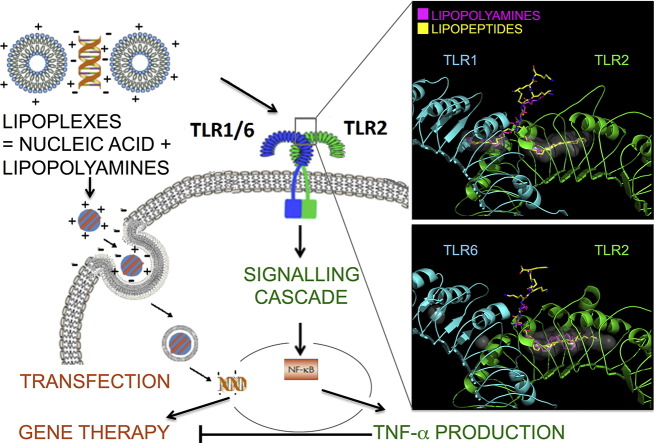

## 1 Introduction

Gene therapy, a technique that aims to replace a defective or missing gene with its normal allele at its natural location, emerged in the 70's [1], with the first successful somatic treatment to leave permanent DNA modification performed in the 90's. Nonetheless the technique is still in its infancy, and remains experimental in treating most diseases that can be traced back to gene disorders. Recently, the European Commission has approved treatment for adult patients diagnosed with familial lipoprotein lipase deficiency (LPLD) and for children with severe combined immunodeficiency due to adenosine deaminase deficiency (ADA-SCID) [2], [3], [4].

The success of gene therapy is conditioned by the development of vectors able to transfect cells efficiently with minimal adverse effects [5], [6], [7], [8], [9]. The most widespread technique of transfection involves viral vectors [10], [11]. Viral vectors exhibit a high efficiency of transfection, but because of their inherent immunogenicity, the risk of gene transmission and/or recombination with germline cells, the limited space for foreign therapeutic genes and the important limitations with respect to scale-up procedures and costs, synthetic alternatives have been proposed [7], [12], [13], [14], [15].

Among available synthetic vectors, cationic lipids, introduced by Felgner in 1987 [16], have been widely studied and commonly used as a result of their relatively high effectiveness, ease of production, lower toxicity and immunogenicity and the possibility to confer tissue specificity [14], [17], [18], [19], [20], [21]. Nevertheless, it was further demonstrated that transfection with cationic lipids/nucleic acids complexes, called lipoplexes, causes inflammatory responses in vitro and in vivo [22], [23], [24], [25]. In the last decade it became apparent that delivery of foreign nucleic acids using cationic lipids maximizes their exposure to pattern recognition receptors (PRRs) located in the endosomal compartment and cytosol with a significant risk of triggering a dangerous immune response and decreasing the transfection efficiency [8], [26]. In particular several endosomal Toll-like receptors (TLRs) are dedicated to nucleic acid recognition: TLR9 recognizes unmethylated CpG motifs of plasmid DNA (pDNA) [27], [28], [29], [30] and TLR7/8 and TLR3 recognize single (ssRNA) and double stranded (dsRNA) RNA, respectively [31], [32]. TLR engagement by nucleic acids activates a signalling cascade leading to translocation of the nuclear factor -κB (NF- κB) into the nucleus, followed by transcription and production of several pro-inflammatory cytokines such as Tumor Necrosis Factor α (TNF-α), Interleukin 6 and 12 (IL-6 and IL-12), which were all reported after administration of lipoplexes [22], [23], [25].

Despite these cytokines being shared by multiple signalling pathways [33], [34] the immuno-stimulatory properties of lipoplexes were generally attributed to activation of TLR3, TLR7/8 and TLR9 by foreign nucleic acids [22], [23], [35]. New approaches to minimize nucleic acid-dependent immune responses were developed. Among them, minicircle DNA, in which bacterial sequences required for production in bacteria but not for gene expression have been removed [36], [37], and CpG-free technologies, that avoid TLR9 activation by using pDNA completely devoid of unmethylated CpG [38], are the most advanced methods. Also several RNA chemical modifications were performed to avoid interaction with PRRs and prevent activation of an immune response [39]. Despite all these efforts, the results were not as successful as expected: although preventing nucleic acids from triggering an immune response does contribute to reducing the inflammation associated with lipoplexes, cytokine secretion has not been eliminated [40], [41], [42], [43], [44]. This suggests that there are other mechanisms responsible for the innate immune responses of lipoplexes, which might be linked to their second component, the cationic lipids.

Cationic lipid nanocarriers were typically considered as inert to the immune system. Recent studies have shown that they are instead involved in several cell-signalling mechanisms either with inflammatory or anti-inflammatory properties [45], [46], [47], [48], [49]. Because of the wide number of structures and variety of cellular effectors that can be involved, the number of cationic lipids that have been investigated so far in terms of their immunostimulatory properties is quite limited [49]. Among cationic lipids, the number of lipopolyamines (LPAs) available is increasing due to ease of synthesis, high transfection efficiency and low toxicity. Indeed spreading the cationic charge with primary and secondary amines improves interaction with nucleic acid and reduces the toxicity associated with the localized cationic charge of tertiary amines and the ether linkage, while the amide linker improves serum compatibility and biodegradability [50], [51], [52], [53], [54], [55].

**Fig. 1:**
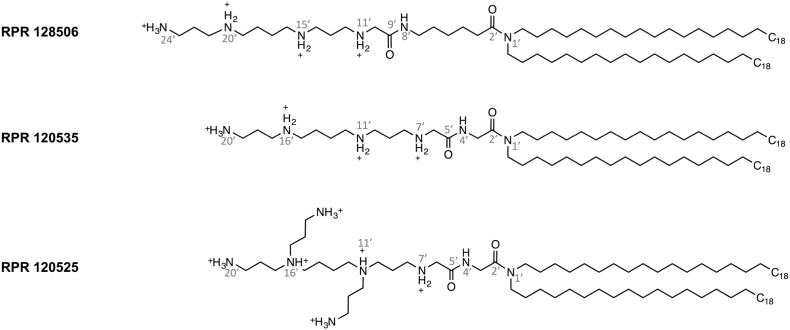
Structures of cationic LPAs tested in this study.

In this paper, we investigate the role of three LPAs (Fig. 1) on the inflammatory processes induced during transfection with lipoplexes. These cationic lipids were previously developed in the context of an intracellular delivery program and successfully used as DNA and siRNA delivery vectors [56], [57], [58], [59]. Our results demonstrate that they all activate TLR2 and confer inflammatory properties to the corresponding lipoplexes. We elucidated the structural parameters that cause TLR2 recognition and compared the LPAs mode of binding to known TLR2 ligands.

## 2 Materials and methods

### 2.1 Reagents and cell lines

Human embryogenic kidney cells were purchased from the American Type Culture Collection (293 [HEK293] (ATCC® CRL1573™)) and human acute monocytic leukemia cell line (THP1 ECACC 88081201) were obtained from European Collection of Authenticated Cell Cultures. RPMI 1640 (Roswell Park Memorial Institute) and DMEM (Dulbecco's Modified Eagle's Medium) media, l-glutamine, sodium pyruvate, penicillin and streptomycin were from Lonza. Phorbol 12-myristate 13-acetate (PMA) was from Sigma Aldrich. Fetal bovine serum (FBS) from South America was from Lonza and FBS from North America was purchased from Sigma-Aldrich. Ultrapure standard lipopolysaccharide (LPS) from *E. coli* 0111:B4, Pam_3_CSK_4_, and Pam_2_CSK_4_ were from InvivoGen. Human TLR2, TLR1, TLR6 and TLR4 neutralizing antibody were purchase from InvivoGen (Cat. Code pab-hstlr2, pab-hstlr1, pab-hstlr6 and pab-hstlr4).

### 2.2 Liposome preparation

RPR120525, RPR120535 and RPR128506 were synthesized as described earlier [45] and stored as powder at − 20 °C. Lipid films were formed by dissolving powder in chloroform, followed by solvent evaporation under nitrogen stream and vacuum drying overnight, and kept at − 20 °C. Liposomes were freshly formed by resuspending lipid films into filtered Hepes 10 mM heated at 56 °C and sonicated for 5 min (BioRuptor, Diagenode) before each experiment.

### 2.3 Lipoplex preparation

The protocol used for lipoplex formation was one described earlier for transfection experiment (6 nmol of cationic lipid per μg of nucleic acids) [59], [60], [61]. Briefly, small interfering RNA (MISSION® siRNA Universal Negative Control from SigmaAldrich Cat. N. SIC001) and plasmid DNA (pcDNA3.1 from Invitrogen Cat. N. V79020) were suspended in 150 mM NaCl and mixed with an equivalent volume of liposomes, then incubated for 20 min at room temperature.

### 2.4 Cell cultures

HEK 293 cells were maintained in DMEM supplemented with 10% heat-inactivated FBS from North America, 2 mM l-glutamine, 1 mM sodium pyruvate, 50 U/mL penicillin, 50 μg/mL streptomycin.

THP-1 cells were cultured in RPMI 1640 supplemented with 10% heat-inactivated FBS from South America, 2 mM l-glutamine, 1 mM sodium pyruvate, 50 U/mL penicillin, 50 μg/mL streptomycin.

All cells were incubated at 37 °C in a 5% CO_2_ atmosphere and tested for mycoplasma contamination on a regular basis. To avoid divergence from the parent line, cell cultures were passaged up to 10 times.

### 2.5 HEK293 cell transfection and stimulation

Cells were transfected as previously described [62]. Briefly, cells were seeded at 4 × 10^4^ cells/mL in 96-well plates (200 μL/ well) and transiently transfected 4 days later. Expression vectors containing a NF-κB transcription reporter vector encoding firefly luciferase (10 ng/well pNF-κB-luc from Clontech), and a constitutively active reporter vector encoding Renilla luciferase (5 ng/well phRG-TK; Promega), together with empty vector (pcDNA3.1 from Invitrogen Cat. N. V79020) and cDNA encoding human membrane CD14 (3 ng/well) and human TLR2 (0.5 ng/well) or human TLR4/MD2 (3 ng/well) (kindly provided by Prof. Clare Bryant, University of Cambridge) were mixed with jetPEI (Polyplus transfection Cat.N. 101-10N) and incubated with cells according to manufacturer's instructions. After 48 h, medium was replaced with serum free medium and cells were incubated for 1 h, cells were additionally washed with serum free medium, then directly incubated for 6 h with tested cationic lipids or lipoplexes (in serum-free medium), ultrapure LPS (100 ng/mL), Pam_3_CSK_4_ (30 ng/mL) or Pam_2_CSK_4_ (30 ng/mL) (in complete medium). Cells were washed with PBS and then lysed with passive lysis buffer (Promega). Luciferase and Renilla activity on cell lysates were quantified on a BioTek Synergy HT microplate reader using home-made luciferase reagent (20 mM Tricine, 2.67 mM MgSO_4_·7H_2_O, 0.265 mM (MgCO_3_)_4_Mg(OH)_2_·5H_2_O, 0.1 mM EDTA, 33.3 mM DTT, 530 μM ATP, 270 μM Acetyl Coenzyme A (Lithium salt), 470 μM luciferin (Biosynth), pH 7.8, diluted 2 times in water before use) or coelenterazine (Biosynth) dissolved in ethanol at 1 mg/mL and diluted 500 times in PBS before use as previously described [63]. Firefly and Renilla luciferase activity on cell lysates were normalized and data were expressed as fold induction as compared to unstimulated conditions.

### 2.6 THP-1 cell stimulation

THP-1 cells were primed by resuspending them in fresh medium containing 50 nM PMA and seeding them in 96-well plates at 3.5 × 10^6^ cells/mL in 200 μL/well two days prior to stimulation. After 24 h of incubation with PMA, the medium was replaced with fresh one, and cells were incubated overnight to further allow cell differentiation.

The day of stimulation, medium was replaced with serum free medium and cells were incubated for 1 h, then washed with serum free medium. When specified cells were incubated for 1 h in the presence of antibodies blocking human TLR2, TLR1, TLR6 or TLR4, at final concentrations of 20 μg/mL in serum-free medium prior to stimulation with liposomes (in serum-free medium), Pam_2_CSK_4_ or Pam_3_CSK_4_ (15 or 30 ng/mL), or ultrapure LPS (100 ng/mL) (in complete medium). Stimulation was carried out over 5 h.

### 2.7 Cytokine assays

After stimulation, cell culture supernatants were collected and assayed for human TNF-α, using DuoSet ELISA kits from R&D Systems, according to manufacturer's instructions with a BioTek Synergy HT Microplate Readers.

### 2.8 Homology modelling

The crystal structure of TLR2/TLR6 bound to Pam_2_CSK_4_ is known for mouse proteins (Protein Data Bank (PDB) code 3A79). A set of twenty human homology models were generated based on the mouse structure using Modeller software 9 version 10 [64]. Among these 20 models, the model with overall lowest discrete optimized protein energy (DOPE) score was chosen for further characterization. The quality of the best model was then compared to the mouse crystal structure at individual amino acid levels with an additional DOPE energy profile, smoothed over a 15 residue window and normalized by the number of restraints acting on each residue. The profile was subsequently visualized in Excel and revealed the adequacy of the model and the template. The quality of the model was good due to the high sequence identity between mouse and human species (about 70% in the region of interest corresponding to residues 33 to 482). As previously reported the shape and size of the lipid binding domains in TLRs vary dramatically between human and mouse [65]. In our TLR2/TLR6 model we estimate that the volume of the pocket increases by approximately 1.6 times in human compared to mouse using CastP software [66].

### 2.9 In silico construction of lipopolyamines

The atomic coordinates of RPR ligands were generated using Sybyl software (Tripos). The geometry of the molecules were optimized using the Powell minimization method with initial optimization based on the Simplex method, and with a gradient of 0.05 kcal/mol and a maximum of 100 cycles of iteration. Partial charges were computed based on the Gasteiger-Hückel charge method. Autodock was used to convert the file into PDBQT format, which contains the atomic coordinates, partial charges and atom types of the molecule.

### 2.10 Molecular docking

The human crystal structure of TLR2 in complex with TLR1 (PDB code 2Z7X) and its homology model in complex with TLR6 were used for docking upon removal of the synthetic ligands bound to the protein complexes. RPR molecules were docked into TLR2 heterodimers using Autodock Vina [67]. The receptor was kept rigid, whereas the ligand was allowed total flexibility. The Autogrid parameters were computed on an initial grid size of 40 × 40 × 50 Å^3^, with a spacing of 1 Å. The grid was centered on the complex at x = + 13.706; y = − 23.174; z = + 3.332. The default optimization parameters were used for the iterated search in Vina, with the default value of 8 for exhaustiveness. Flexible docking performed with LPAs generated a number of poses ranked according to their binding energies. The first 9 poses were then resubmitted to rigid docking in order to compare the binding energy of molecules of different sizes. This extra step was found useful to circumvent the biased scoring function in Autodock Vina dependent on the number of torsion angles. Docking poses were analysed and structural images were generated in PyMol (http://www.pymol.org) and Chimera [68]. Detailed interactions were also analysed using LigPlot + [69].

## 3 Results

### 3.1 Lipoplexes induce lipopolyamine-dependent TNF-α secretion in human macrophages

In order to investigate the role of cationic lipids in the inflammatory processes induced during transfection, we studied the immunostimulatory properties of lipoplexes and liposomes made with RPR 128506, 120535 and 120525 (Fig. 1), cationic lipopolyamines (LPAs) successfully used as DNA and siRNA delivery vectors [56], [57], [58], [59], [60], [61], whose polar head is constituted by polyamines mimicking peptide backbones linked to aliphatic hydrocarbon chains by an amide group. All three carry diC18:0 fully saturated aliphatic chains but differ in their cationic head group structure. RPR 128506 and 120535 are linear with one terminal primary amine and three secondary amines. RPR 128506 contains a pentane linker region between both its amide bonds, whereas RPR 120535 and 120525 have a methane group instead. RPR 120525 is branched at N11′ and N16′, thus bearing three terminal amines and two tertiary ones.

**Fig. 2:**
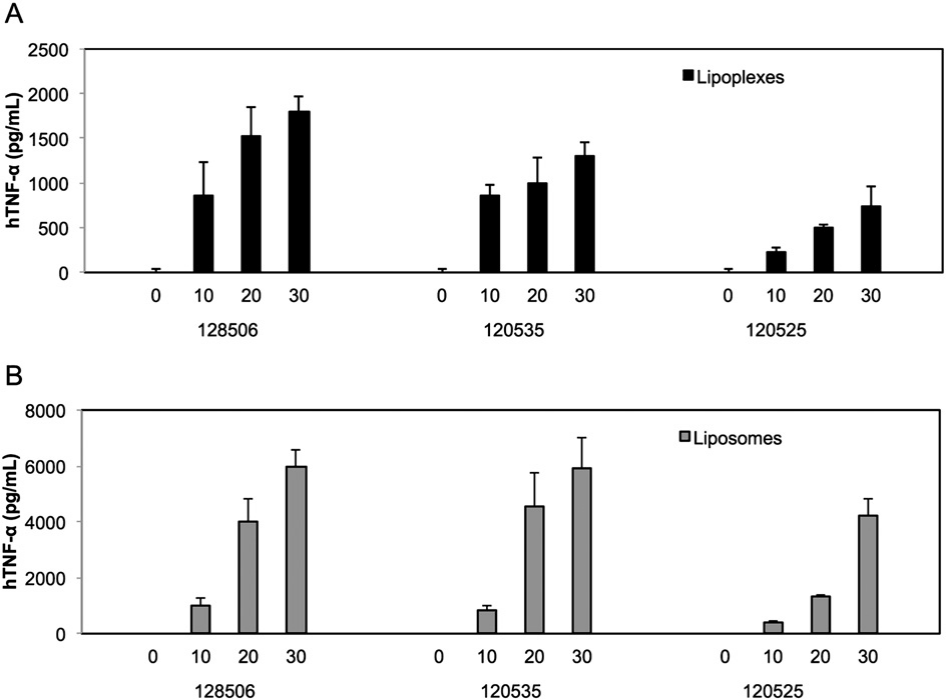
Lipoplexes (A) and liposomes (B) lead to TNF-α secretion in human macrophages. Primed THP1 cells were incubated for 5 h with the indicate amount of cationic lipids (μM) in serum free medium, then cell supernatants were collected and TNF-α was quantified using ELISA assay following manufacturer's instructions. Each bar represents the mean + standard deviation of three replicate values (*n* = 3) after subtraction of the secretion measured in absence of stimulant. The experiment is representative of at least 3 independent replicates.

In order to characterize the inflammatory reaction induced by lipoplexes and to investigate the involvement of LPAs rather than nucleic acids we measured TNF-α secretion in primed THP-1 cells (a monocytic cell line that expresses the whole repertoire of TLRs), after incubation with increasing amounts of RPR 120535, 120525 or 128506 bound to nucleic acids (lipoplexes) or not (liposomes). As shown in Fig. 2, lipoplexes made from RPR 120535, 120525 and 128506 induce a dose-dependent production of TNF-α cytokine and this increased in the absence of nucleic acids cargo.

### 3.2 TLR2 activation by lipopolyamines is responsible for lipoplex-induced TNF-α secretion

**Fig. 3:**
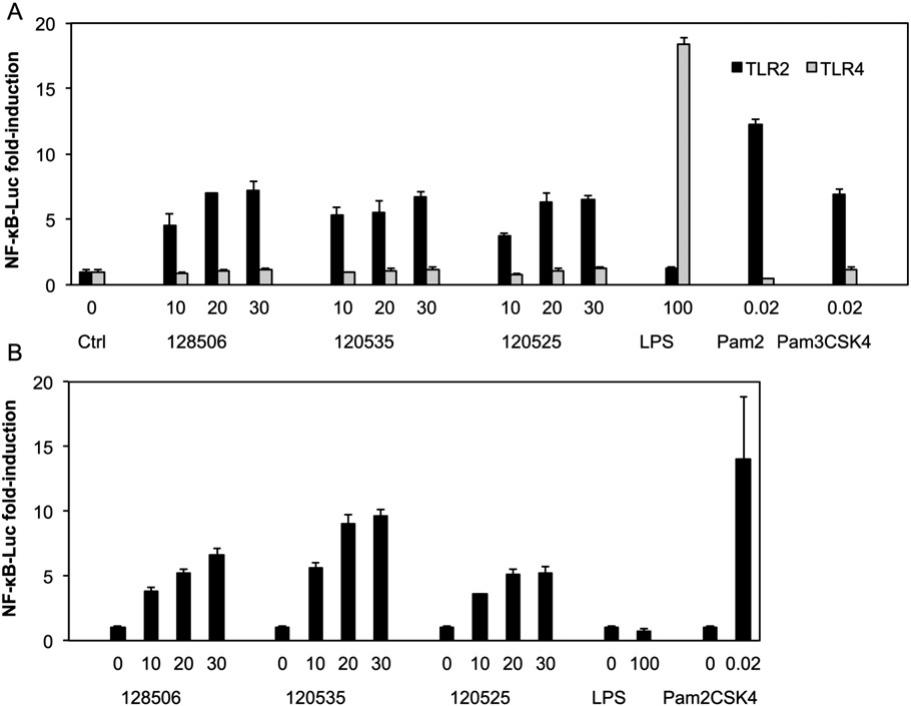
Liposomes (A) and lipoplexes (B) activate NF-κB through a mechanism dependent on TLR2. HEK 293 cells were transfected with plasmids encoding hTLR2 and hCD14 (black), or hTLR4, hMD2 and hCD14 (grey) together with a luciferase reporter plasmid dependent on NF-κB activation. Two days after transfection, cells were incubated for 1 h in serum-free medium, washed and then incubated for 6 h with serum free medium alone (Ctrl, 0) or the indicate amount of liposomes or lipoplexes (μM, serum free medium) or LPS (ng/mL, complete medium) or Pam_2/3_CSK_4_ (μM, complete medium). Luciferase and Renilla were then quantified in cell lysate, normalized and reported here as fold induction as compared to control. Each bar represents the mean + standard deviation of three replicate values (*n* = 3). The experiment is representative of at least 3 independent replicates.

In order to identify the receptor responsible for the immunostimulatory properties of our LPAs, we used a human embryonic kidney 293 (HEK293) cell line deficient in most TLRs, except for TLR1 and TLR6 [70], [71], [72], and introduced the two human TLRs that recognize lipids, Toll-like receptors 2 or 4, by transient transfection with their co-receptors CD14 or MD2 and CD14 respectively. We observed NF-κB activation after incubation with liposomes in a dose-dependent manner when HEK293 cells were transfected with hTLR2/CD14 (black bars), in contrast to cells transfected with hTLR4/MD2/CD14 (grey bars) (Fig. 3A). As expected, no activation was detected after stimulation of hTLR2/CD14 transfected cells with the TLR4 ligand, lipopolysaccharide (LPS), whereas stimulation with the TLR2/TLR1 specific ligand, the synthetic lipopeptide Pam_3_CSK_4_, and the TLR2/TLR6 specific ligand, Pam_2_CSK_4_ led to robust NF-κB activation. Similarly, hTLR4/MD2/CD14 transfected cells responded only to LPS and not to TLR2 ligands.

In order to investigate if LPAs are able to activate TLR2 even when complexed to nucleic acids, we incubated TLR2-transfected cells with lipoplexes formed by incubating RPR 120535, 120525 or 128506 with siRNA and pDNA as described in the transfection protocol [56], [57], [58], [59], [60], [61]. As with the liposomes, lipoplexes activated NFκB when incubated with TLR2/CD14-transfected HEK293 cells. As expected these cells responded only to Pam_2_CSK_4_ and not to TLR4 ligands (Fig. 3B).

**Fig. 4:**
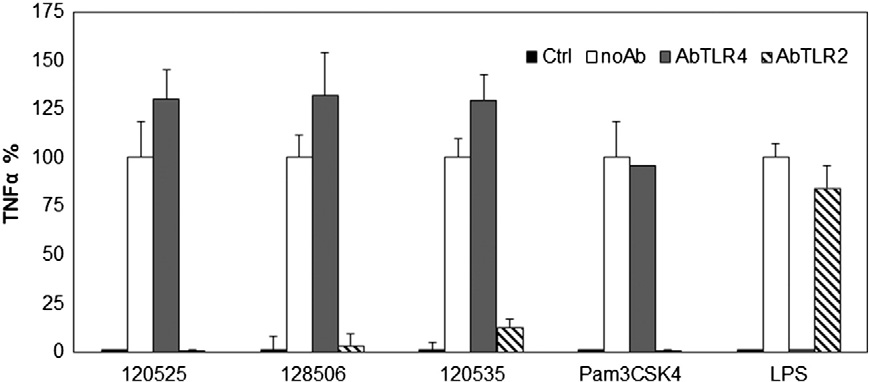
Lipoplex-induced TNF-α secretion is solely dependent on TLR2. Primed THP1 cells were incubated for 1 h in serum-free medium, then 1 h with or without (noAb) 40 μg/mL (20 μg/mL final concentration after addition of stimulants) antibodies blocking TLR2 (AbTLR2) or TLR4 (AbTLR4). Then cells were incubated for 5 h with serum free medium alone (Ctrl) or with RPR 120535, 120525 and 128506 made lipoplexes (20 μM, serum free medium) or LPS (100 ng/mL, complete medium) or Pam_3_CSK_4_ (30 ng/mL, complete medium). The supernatant was collected and TNF-α quantified by ELISA. Data are expressed as percent of secretion as compared to condition without antibodies (no anti) for each stimulant after subtraction of control. Each bar represents the mean + standard deviation of three replicate values (*n* = 3). The experiment is representative of at least 3 independent replicates.

Next we tested whether activation of TLR2 contributed significantly to TNF-α secretion induced by RPR lipoplexes. This was achieved by pre-incubating primed THP-1 cells with antibodies that block hTLR2 or hTLR4 prior to stimulation with lipoplexes. The stimulatory effect of lipoplexes, measured through TNF-α secretion in cell supernatants, was totally inhibited by antibodies blocking TLR2 (Fig. 4), demonstrating the role of TLR2 activation in the immunostimulatory properties of LPA lipoplexes. The absence of inhibition after pre-incubation with antibodies blocking TLR4 demonstrates that the effect observed with *anti*-TLR2 is specific. Controls with LPS and Pam_3_CSK_4_ confirmed that the antibodies were effective and specific at the concentrations used.

### 3.3 LPA-induced TNF-α secretion is mainly dependent on TLR2/TLR1 heterodimer

**Fig. 5:**
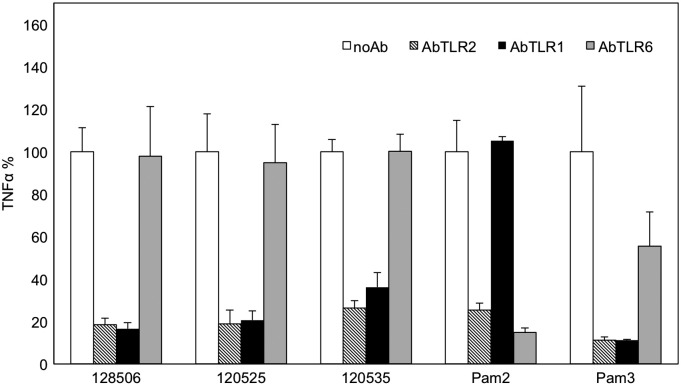
LPA-induced TNF-α secretion is mainly dependent on TLR2/TLR1 heterodimer. Primed THP1 cells were incubated for 1 h in serum-free medium, then 1 h with or without (no Ab) 40 μg/mL of antibodies blocking TLR2 (AbTLR2), TLR1 (AbTLR1) or TLR6 (AbTLR6). Then cells were incubated for 5 h with RPR 120535, 120525,128506 (20 μM, serum free medium), Pam_2_CSK_4_ or Pam_3_CSK_4_ (15 ng/mL, complete medium). The supernatant was collected and TNF-α quantified by ELISA. Data are expressed as percent of secretion as compared to condition without antibodies (noAb) for each stimulant. Each bar represents the mean + standard deviation of three replicate values (*n* = 3). The experiment is representative of at least 3 independent replicates.

In order to identify which co-receptor is involved in TLR2 activation by LPAs, we pre-incubated primed THP-1 cells with antibodies blocking hTLR2, hTLR1 or hTLR6 before stimulation with LPA liposomes, the TLR2/TLR1 specific ligand Pam_3_CSK_4_, or the TLR2/TLR6 specific ligand Pam_2_CSK_4_. TNF-α secretion induced by LPAs was strongly inhibited by antibodies that block TLR2 and TLR1 (Fig. 5), demonstrating the main role of TLR2/TLR1 heterodimer in the immunostimulatory properties of LPAs. Controls with Pam_2_CSK_4_ and Pam_3_CSK_4_ confirmed that the antibodies were effective and specific at the used concentrations.

### 3.4 RPR lipopolyamines bind TLR2 heterodimers with affinities comparable to lipopeptides

In order to better understand the mechanism of action of LPAs we performed docking studies on TLR2 alone and in complex with TLR1 or TLR6. We compared their binding mode and strength to available crystal structures. We found that RPR molecules bound with greater affinity to heterodimeric rather than to monomeric TLR2 in support of their agonistic activity. For instance, RPR 120535 binds the inactive conformation of TLR2 with a binding energy of − 27.6 kcal/mol and the active conformations of TLR2 at − 28.5 to − 32.5 kcal/mol in complexes with either TLR6 or TLR1, respectively (Table 1). Moreover all three RPR molecules tested bound both TLR2/TLR1 and TLR2/TLR6 heterodimers with binding energies around − 30 kcal/mol.

**Table 1:** Binding energies and polar interactions between ligands and TLR2 heterodimers. Conserved interactions compared to known crystal structures are underlined. *Pam_2_CSK_4_ is bound to mouse proteins. All other models involve human proteins.

	Binding mode		Binding energy	TLR2		TLR1		TLR6	
				Main chain	Side chain	Main chain	Side chain	Main chain	Side chain
RPR 120535	TLR2/1	Both chains in TLR2	− 31.1 kcal/mol	F325 F349 L350					
		One chain in each receptor	− 32.2 kcal/mol	L324 F325	N294 S329	G313′			
	TLR2/6		− 28.5 kcal/mol	F325 Y326 L350 F349					
RPR 128506	TLR2/1	Both chains in TLR2	− 31.9 kcal/mol	L324 F325 F349 L350		F312	Q316′		
		One chain in each receptor	− 31.4 kcal/mol	D327		G313′	Q316′		
	TLR2/6		− 29.9 kcal/mol	L324					
RPR 120525	TLR2/1	Both chains in TLR2	− 32.1 kcal/mol	L324 F325 S346					
		One chain in each receptor	− 32.5 kcal/mol	F325	D327 S329 Y326	G313′Q316′			
	TLR2/6		− 31.7 kcal/mol	G293 F325				L318′	
Pam_2_CSK_4_*	TLR2/6		− 25.2 kcal/mol	F325 D327 F349				F319′	
Pam_3_CSK_4_	TLR2/1		− 35.4 kcal/mol	F349	N294	G313′	Q316′		

In an attempt to rationalize these energy terms we also calculated them for TLR2 ligands with known crystal structures. We used the same protocol to compare directly the values obtained for the LPAs tested with the lipopeptide ligands. Pam_3_CSK_4_ binds human TLR2/TLR1 with much higher affinity at − 35.4 kcal/mol. Pam_2_CSK_4_ bound mouse TLR2/TLR6 at − 25.2 kcal/mol. Hence, RPR 120535, RPR 120525 and RPR 128506 seem to be able to bind TLR2 heterodimers as strongly as lipopeptides characterized by crystallographic studies.

### 3.5 RPR lipopolyamines adopt docking poses reminiscent of lipopeptide binding mode

**Fig. 6:**
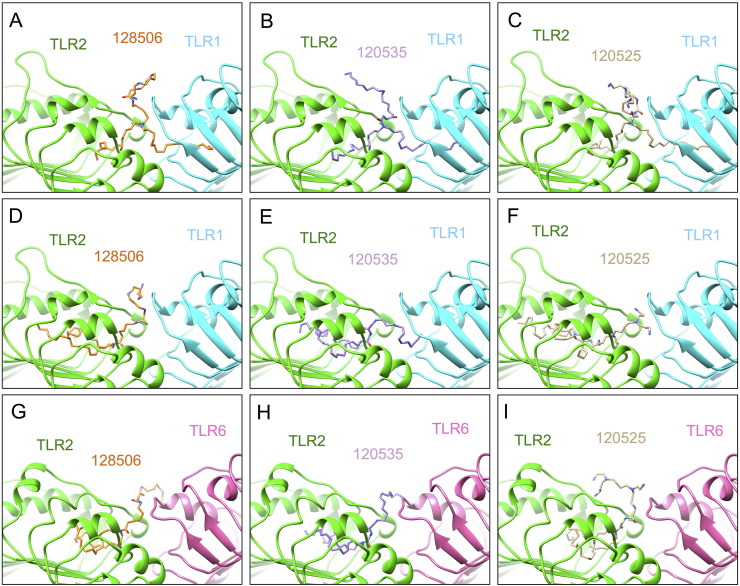
Docking poses of di-C18 lipopolyamines. Three binding modes are predicted: (A–C) binding to TLR2/TLR1 heterodimer with one chain buried per TLR pocket; (D–F) with both chains buried into TLR2; (G–I) binding to TLR2/TLR6 heterodimer with both chains buried into TLR2. The cartoon representation of TLR complexes was generated by Chimera.

Visualization of the docking poses of the RPR molecules revealed 3 types of binding modes for each lipid: (i) binding to TLR2/TLR1 with one aliphatic chain in each TLR (Fig. 6 A-C); (ii) binding to TLR2/TLR1 with both chains in TLR2 (Fig. 6 D-F); (iii) binding to TLR2/TLR6 with both chains in TLR2 (Fig. 6 G-I).

**Fig. 7:**
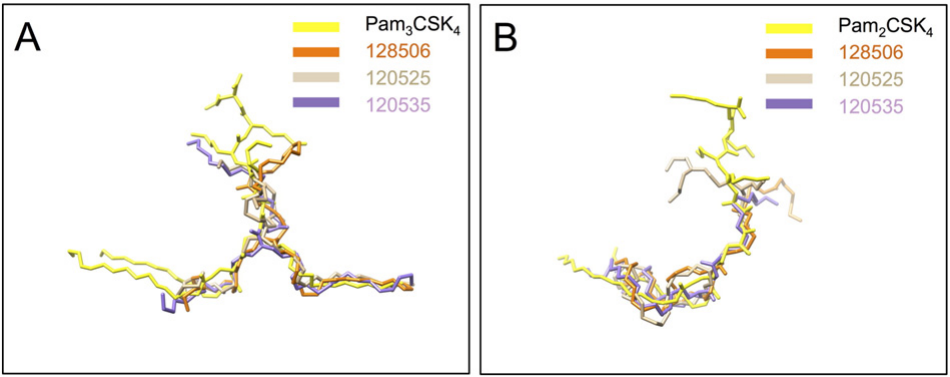
LPA binding modes are reminiscent of bacterial lipopeptide recognition. (A) Superposition of LPAs in the highest affinities complexes with the crystal structure of triacylated C16 lipopeptide Cys-Ser-Lys_4_ (Pam_3_CSK_4_) bound to human TLR2/TLR1 (not shown). (B) Superposition of LPAs with the crystal structure of diacylated C16 lipopeptide Cys-Ser-Lys_4_ (Pam_2_CSK_4_) bound to mouse TLR2/TLR6 (not shown). This figure has been generated in PyMol using aligned protein structures or homology models. The ligands are represented in coloured sticks.

**Fig. 8:**
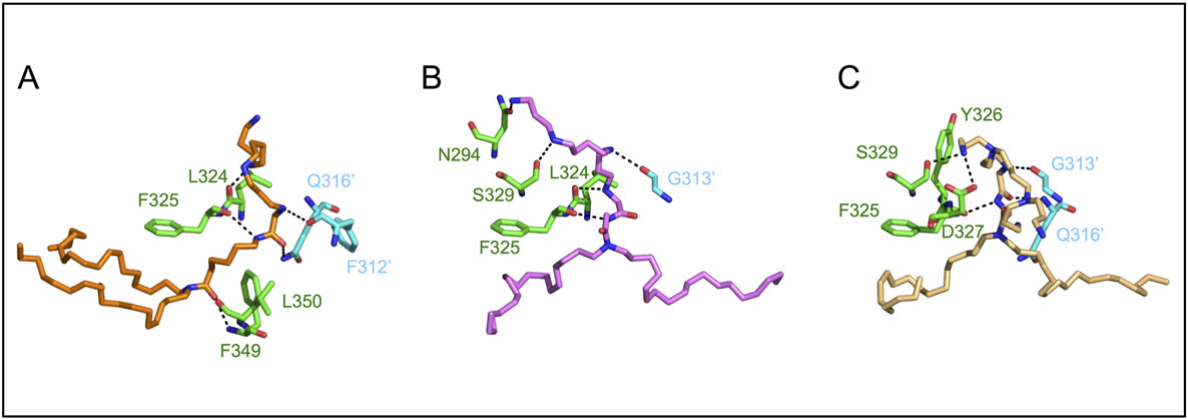
Hydrogen bonds in LPA-TLR2 complexes are mainly mediated by the peptide chain instead of side chains. Polar contacts in the highest affinity LPA complexes. (A) RPR 128506; (B) RPR 120535; (C) RPR 120525. TLR2 residues involved in hydrogen bonds are shown in green and TLR1 in cyan.

All poses show a degree of similarity to lipopeptides Pam_2_CSK_4_ and Pam_3_CSK_4_ binding as suggested by their superposition (Fig. 7). LPAs occupy the hydrophobic pocket of TLR2 either partially or fully and form conserved and new hydrogen bonds compared to known crystal structures with residues from TLR2 leucine-rich repeat (LRR) 11 to 13 and TLR1 or TLR6 region spanning LRR9 to 12 (Table 1, Supplemental Figs. S1–S9). It is also remarkable that, as in the crystal structures, most hydrogen bonds are generated with the TLR peptide backbone (Fig. 8, Table 2).

**Table 2:** Hydrogen bonds in TLR2-liganded complexes are mostly mediated by the peptide chain. The table lists the number of hydrogen bonds with the main chain (first number) and the side chains (second number). n.d. stands for not determined. *Pam_2_CSK_4_ is bound to mouse proteins. All other models involve human proteins.

	TLR2/TLR1		TLR2/TLR6
Lipid chains binding mode	Both chains in TLR2	One chain in each receptor	Both chains in TLR2
RPR 120535	3/0	3/2	4/0
RPR 128506	5/1	2/1	1/0
RPR 120525	3/0	3/3	3/0
Pam_2_CSK_4_*	n.d	n.d	4/0
Pam_3_CSK_4_	n.d	2/2	n.d

As with lipopeptide ligands, LPAs are able to occupy the TLR1 pocket with one fully extended lipid chain while engaging partially the TLR2 pocket with their second chain (Fig. 7A). Indeed the bend conformation within the hydrophobic TLR2 cavity allows an individual aliphatic chain to fill an otherwise much deeper pocket. This conformation is similar to a cis double bond in an unsaturated chain between atoms C7 and C10. Superposition of the docking poses shows that Pam_3_CSK_4_ projects carbon atoms 9 to 11 of its lipid chains deeper inside TLR2 compared to the U-turn adopted by RPR molecules (Fig. 7A). Moreover this is not the preferred binding mode of the more hydrophobic compound, RPR 128506, that favours a more buried conformation with both aliphatic chains inside TLR2 (Fig. 6D). The preference of this compound for a TLR1 instead of a TLR6 heterodimer will be discussed.

Overall it is striking how well the conserved amide-bound lipid chains of all RPR molecules overlap with Pam_3_CSK_4_ in their TLR1 binding mode, whereas TLR2 binding seems to offer more flexibility, both at the level of their lipid tail and cationic headgroup positioning (Fig. 8). While all docking poses are shown in Supplemental Figs. S1–S9, below we will only describe at an atomic level one docking pose to illustrate for each lipid the complex of highest affinity. We use a standard numbering for TLR2 residues whereas TLR1 amino acids will be referred to with an asterisk, TLR6 amino acids, with two asterisks and conserved contacts compared to crystals are discussed.

### 3.6 RPR 120535 and 120525 bind preferentially TLR2/TLR1 with one lipid chain per TLR

The highest affinity complex is formed by linear RPR120535 and its branched counterpart RPR 120525, which bound with energies of − 32.2 and − 32.5 kcal/mol, respectively, with TLR2/TLR1 with one lipid chain per TLR (Fig. 6 B–C, Table 1 and Supplemental Figs. S2 and S3).

One lipid chain of RPR 120525 makes hydrophobic interactions with TLR2 residues from LRR11 (Ile319, Leu324, Tyr326, Leu328), LRR12 (Val343, Lys347, Val348, Phe349, Leu350, Val351, Pro352, Leu355) and LRR13 (Tyr376). TLR1 buries the other one using residues from LRR9 (Trp258′), LRR11 (Ile304′, Val311′, Phe312′, Phe314′, Pro315′, Ile319′, Tyr320′, Phe323′) and LRR12 (Thr336′, Arg337′, Met338′). The polyamine headgroup makes hydrophilic interactions with TLR2 residue Phe325 in LRR11 via the carbonyl group of its peptide chain, which is a hydrogen acceptor of the secondary amine in position 4′. TLR2 also makes unique side chain interactions, in particular an ionic interaction between primary amine N15” and the carboxylate side chain of Asp327, along with a hydrogen bond between the same amine, Y326 and Ser329.

Crosslinking interactions are mediated between the polyamine headgroup and the peptide chain of TLR1 at Gly313′ and Gln316′ from LRR11. The interaction with Gly313′ occurs between its CO group (hydrogen bond acceptor) and the ligand′s secondary amine at N7′ (hydrogen bond donor) and is shared by Pam_3_CSK_4_ that binds in a similar fashion. In contrast the contact mediated by Gln316′ differs between the two ligands. While Gln316′ mediates a side chain amine NE2 interaction in the Pam_3_CSK_4_ complex, here the interaction is mediated instead by its main chain amino group as a hydrogen bond donor to the ketone C =O in position 5′ of RPR 120525 (Fig. 8C).

According to our docking analysis RPR 120535 also binds preferentially with its lipid chains spread across the TLR2/TLR1 heterodimer despite its binding mode differing quite significantly at the level of hydrogen bonding (Fig. 8B). In contrast to its branched counterpart this molecule is only a hydrogen bond donor with each amine group involved. Its terminal amine N20′ gives a hydrogen bond to the OD1 side chain of TLR2 Asn294. Next secondary amine N20′ interacts with the hydroxyl group of Ser329 and N15′ contacts TLR1 Gly313′ at the dimeric interface. N11′ shares 2 hydrogen bonds with the peptide chain at TLR2 Leu324 and Phe325 that also reaches out to N8′. Hydrophobic contacts overlap well with the ones described previously and involve TLR2 LRR11 residues (Ile319, Phe322, Tyr326, Asp327, Thr330), LRR12 (Val343, Lys347, Val348, Phe349, Leu350, Val351, Leu355) and LRR13 residues (Leu359, Leu365, Leu367, Tyr376); and TLR1 LRR9 residue (Phe261′), LRR11 (Ile304′, Val307′, Phe312′, Phe314′, Pro315′, Gln316′, Ile319′, Tyr320′) and LRR12 (Thr336′, Arg337′).

### 3.7 RPR 128506 binds preferentially TLR2/TLR1 with both chains in TLR2

RPR 128506 docks best to TLR2/TLR1 by inserting both lipid chains in TLR2 instead of one chain per TLR (Fig. 6 D, Table 1 and supplemental S4). This compound is similar to RPR 120535 in structure with its linear head group but contains a linker of an additional four carbons between its amide groups, which seems to improve its binding to TLR2 within the TLR2/TLR1 heterodimer compared to RPR 120535. The hydrophobic spacer may also deter binding of one chain per TLR due to its entropic contribution and adverse solvent exposure.

In the TLR2/TLR1 complex with both chains occupying TLR2, hydrophobic interactions are mediated by TLR2 LRR9 (Ile261, Leu266), LRR10 (Phe284, Leu289, Phe295), LRR11 (Pro306, Leu312, Ile314, Leu317, Ile319, Tyr326, Asp327, Leu328, Leu331), LRR12 (Leu334, Thr335, Val338, Ile341, Val343, Ser346, Val348) and LRR13 (Leu359, Leu367) and remarkably few TLR1 residues in LRR11 (Phe312′, Gly313′, Pro315′). Two hydrogen bonds are formed with the TLR1 main chain carbonyl group at Phe312′ and the side chain of Gln316′, which is conserved in Pam_3_CSK_4_ binding (Fig. 8A). Another four hydrogen bonds occur with the peptide chain of TLR2 at Leu324, Phe325, Phe349 and Leu350 (LRR11–12) with the CO group of the first two residues and the NH group of the latter two. In the best docking poses the ligand is buried 2.5 Å deeper inside the hydrophobic TLR2 pocket in the TLR1-heterodimer compared to the TLR6 one. This results in an increase in hydrophobic contacts and a decrease of conserved hydrophilic interactions of the headgroup. Indeed none of the usual residues involved in hydrogen bonding are found in the TLR2/TLR6 heterodimer (TLR2 residues Leu324 and Tyr326; TLR1 residues Ser320′, Thr322′ and Tyr323′), which would suggest a novel binding mechanism based on the promiscuous accommodation of the polar head for a ligand with an increased hydrophobic contribution (Table1 and supplemental Figs. S7).

### 3.8 Molecular reason for the TLR2/TLR1 preference over TLR2/TLR6 by LPAs

Overall, despite LPAs possessing two alkane chains, these molecules performed best on TLR2/TLR1 heterodimers in our docking analysis. In order to determine the molecular reason for this preference we looked into the atomic details of RPR 120525, which was the strongest TLR2/TLR6 ligand among the set of LPAs tested at − 31.7 kcal/mol.

In the TLR2/TLR6 complex hydrophobic interactions are mainly mediated by TLR2 residues in LRR9 (Ile261), LRR10 (Phe284, Asn294, Phe295, Arg296), LRR11 (Ile314, Leu317, Ile319, Tyr326, Asp327, Leu328, Ser329, Thr330, Leu331), LRR12 (Ile341, Val343, Ser346, Lys347, Val348, Phe349, Leu350, Val351, Pro352, Leu355) and LRR13 (Leu359, Leu365, Leu367). TLR6 Leu318” and Phe319” contribute to hydrophobic interactions. The former is also a hydrogen bond acceptor via its peptide bond for a primary amine side chain of the LPA. Further hydrogen bonding involves TLR2 residues at Gly293 and Phe325 equally mediated by their main chain CO group. While each ligand has slightly different contacts with the receptor complex, branched RPR 120525 shares more features with Pam_2_CSK_4_ than the other two LPAs (Fig. 7B, Table 1). The atoms that link the lipid chains in both molecules are only 1.0 Å apart with both the headgroups and the lipid chains occupy the same overall space. In contrast, when we compare RPR 120525 in the TLR6 heterodimers to the one bound to TLR1 it appears that the headgroup is shifted upwards by 5.1 Å upon TLR1 interaction. Hence the TLR6 complex conformation may appear more compact compared to the TLR1 with increased TLR2 hydrophobic interactions while preserving a single hydrophilic contact at the dimer interface with TLR6. Given that the TLR1 heterodimer mediates twice as many hydrogen bonds compared to TLR6, the docking algorithm used in this study predicts a higher affinity for the latter (Table 1).

## 4 Discussion

To date TNF-α secretion observed after stimulation of cells with lipoplex has been attributed to activation of TLR3, TLR7, TLR8 and TLR9 by nucleic acids, whereas cationic lipids were considered as inert for the immune system [22], [23], [35]. We presently show instead that the RPR 120525, 120535 and 128506 lipoplexes possess immunostimulatory properties conserved in the absence of cargo nucleic acids, and that di-C18 lipopolyamines RPR 120525, 120535 and 128506 were all able to induce TNF-α secretion through a mechanism strictly dependent on TLR2.

It has been shown that the length of the aliphatic hydrocarbon chains between C12 and C18 in cationic lipids determines their transfection efficiency. The configuration of their linker and head group is equally important; cationic lipids whose aliphatic chains are linked to polyamines by an amide linker show the best transfection efficiency [51], [52], [54], [59], [73], [74].

For these reasons, syntheses of transfection agents have produced, and continue to produce, cationic lipids sharing many features with the LPAs here investigated (the amide linker, the carbonyl and amino groups and the saturated carbon chains) [53], [57], [60], [75], [76], [77], [78], [79], [80], [81].

TLR2 senses a wide range of ligands that includes bacterial lipopeptides and lipoglycans among others [34]. Synthetic LPAs, engineered to deliver nucleic acids to cells, are chemically distinct from typical TLR2 ligands but share these features with lipopeptides.

Molecular docking shows that the alkyl chains of lipopolyamines are shielded from the solvent in a hydrophobic pocket upon TLR2 binding, which was shown to be large enough to accommodate up to 18 carbon atoms [65], [82]. The hydrophilic head imitates carboxyl and amino groups of lipopeptide backbone, which rather than side chains mediate H-bond with TLR residues [65], [82]. Overall, each lipopolyamine forms conserved and new H-bonds, most of which, as in crystals, are with the TLR peptide chains; this lack of specificity is potentially critical for mediating promiscuity in TLR2-ligand sensing.

Thus these features which enhance transfection efficiency also confer immunostimulatory properties to these cationic lipid nanocarriers by mimicking TLR2 ligands. Our results are in agreement with TLR2- lipopeptides structural activity relationships reported by Buwitt-Beckmann [83], Spohn [84], Okusawa [85] and Morr [86], which show that human TLR2 is able to recognize a large variety of synthetic lipopeptide structures, which can differ in terms of chain length (from C12 to C18), peptide sequence and number of amino acids (confirming the promiscuity of the hydrogen bonds). Spohn et al. also showed that lipopeptides in which the S atom of the acrylate cysteine is replaced by a C atom are still able to activate TLR2 but with a reduced biological activity [84]. This substitution is also found in lipopolyamines, and most likely contributes to the weaker maximal activity of RPR compounds compared to lipopeptides (Supplemental Fig. S10).

In agreement with our results, with the aim to confer to a TLR2 ligand a carrier property, InvivoGen replaced the peptide head of lipopeptide Pam_2_CSK_4_ with a polyamine (spermine) and this modification conferred delivery properties without losing TLR2 activation [87]. Here we show that it is due to the promiscuity of H-bond formation, and that TLR2 activation is retained not only when the polar head is replaced by polyamines, but also acyl chains and cysteine linker of lipopeptides by simple alkyl chains.

The di-C14 RPR206252 was so far the only cationic lipid shown to activate Toll-like receptor 2 and only in the absence of nucleic acids [45]. Our work illustrates that the TLR2-dependent immunostimulatory properties previously shown for this lipopolyamine are shared by a set of longer LPAs up to di-C18.

Our results suggest that all saturated LPAs may activate TLR2. This could explain the LPA-lipoplex dependent inflammatory responses observed in lipoplexes deficient in nucleic acid-TLR recognition [40], [41], [42], [43], [44]. However the immune response is dose dependent and is lower when LPAs are in lipoplex form. Furthermore, the response can vary depending on the cell lines or in vivo substrates used. We recommend testing the inflammatory properties of a lipoplex in the conditions used for transfections in order to evaluate its safety.

### 4.1 A hydrophobic tail tale

The two aliphatic chains in LPAs fully occupy the TLR2 lipid-binding pocket and could therefore constitute ideal TLR2/TLR6 ligands. LPAs also share the amide linkage of the third lipid chain of the prototypical lipopeptide Pam_3_CSK_4_. TLR2/TLR1 complexes bound to LPA either display an empty TLR1 pocket and a fully filled TLR2 cavity, or a TLR1 pocket filled with one aliphatic chain and a TLR2 cavity partially filled with one kinked chain which mimicks the binding of two chain. Both cases are entropically unfavourable, which might explain the reduced efficacy and potency of LPAs compared to lipopeptides (Supplemental Fig. S10).

Together with the biological data obtained by blocking TLR1 or TLR6 on human macrophages, the apparent affinities of RPR molecules for TLR2 suggest that LPAs bind preferentially to TLR2/TLR1 but do not rule out TLR6 as a heterodimerization partner of TLR2 in lipopolyamine signalling.

The participation of TLR6 in dimer formation could occur in other cells or conditions, perhaps in the absence of TLR1. Indeed Pam_2_CSK_4_, specific of TLR2/TLR1 heterodimer, has been shown to activate TLR2/TLR6 in knock-out mice or SW620 cells transfected with TLR2 and TLR6 suggesting partially overlapping binding capacities of both types of heterodimers [83], [88].

In the absence of crystallographic data, the actual conformation of an “empty” TLR1 is unknown, in contrast to TLR2, which undergoes structural rearrangements within its lipid binding region located in leucine-rich repeat LRR10–11 [82]. Finally the apparent ligand affinities that we calculated for TLR2/TLR1 and TLR2/TLR6 complexes do not take into account the compensatory interactions made by TLR6 that increases its protein-protein contacts at the dimer interface. In contrast TLR1 relies on the ligand to crosslink the heterodimer. A molecular collaboration between LPAs within a given TLR2 dimer, whether it is with TLR1 or TLR6, might govern the final positioning of the ligand, thus embracing the potential existence of both types of heterodimeric complexes.

Interestingly, in the configuration that RPR bind TLR2/TLR1 heterodimers by crosslinking them with one lipid chain per TLR, it is striking how well the amide bound lipid buried in TLR1 overlays with the one linked to the N-terminal cysteine found in Pam_3_CSK_4_. The overall conformation is more surface exposed compared to exclusively TLR2 bound lipids. A parallel can be drawn with the recognition of underacylated lipopolysaccharide (LPS), lipid IVa, known to trigger activation of mouse TLR4 but not of human TLR4 [89], [90], [91], [92], [93]

In the human complex the ligand is completely buried inside the hydrophobic pocket of MD-2. In the mouse complex, the negatively charged phosphates of the lipid IVa head group contact species-specific positively charged TLR4 residues, which promote an alternative more surface exposed orientation of the ligand [93]. This in turn allows lipid IVa to occupy nearly the same space as LPS, although it lacks two secondary acyl chains. Thus, insufficient hydrophobic interactions by an underacylated ligand can be compensated for by additional polar contacts mediated by the head group. We propose that this principle may also apply to TLR2/TLR1 heterodimers, and propose that diC18 LPAs can activate it with only one aliphatic chain per TLR.

The alkane chain within the TLR2 pocket adopts a U-turn from about *C*4 up to C10 depending on the lipopolyamine. The critical hydrophobic interactions are therefore maintained with one bend aliphatic chain instead of two extended lipid chains thanks to a molecular kink, which occupies the space of two C6 chains approximately. However, separately 2 hexyl chains do not trigger TLR2 activity as described by Buwitt-Beckmann et al. who characterized the TLR2 activity of a PamHex2 lipopeptide [83]. We postulate that the single C18 chain bends in such a manner to block off the entrance of the TLR2 pocket, which seems to generate sufficient steric constraint to activate TLR2. The configuration obtained in the docking pose would be best captured using a cis double bond derivative within one of the alkane chains involving a couple of adjacent carbon atoms located somewhere between C4 and C10. Preliminary results with a new lipopolyamine RPR 208484 that carries one saturated C18 chain and one unsaturated C18:1, Δ9 chain displays the same immune-stimulatory activity as its parent molecule, the fully saturated RPR 120535 (Supplemental Fig. S11), which strongly supports the proposed binding model.

Lipoteichoic acids (LTAs) have been reported to either activate TLR2/TLR1 or TLR2/TLR6 heterodimers [94], [95], [96].

Remarkably, *Streptococcus pneumonia* LTA carries a cis double bond C16:1, Δ9 in only one of its palmitate chain. It is still recognized by TLR2, and activates TLR2/TLR1 although with lower efficiency compared to unsaturated LTA from *Staphylococcus aureus* [95]. In turn, saturated *Staphylococcus aureus* LTA preferentially activates TLR2/TLR6 in a more shielded binding mode that can be enhanced by the cooperative crosslinking of hydrophilic mannose binding lectins [94]. Our LPAs and in particular the branched RPR 120525 which buries itself deeper into the TLR2/TLR6 complex compared to TLR2/TLR1, therefore also compare well with the activity of natural TLR2 ligands.

Moreover given the size of the TLR2 and TLR1 lipid pockets we predict that it is inconceivable for two unsaturated chains to be bound by TLR2 complexes of any kind. This in turn is supported by the total lack of NF-κB dependent immunostimulatory activity of DOSPA component of lipofectamine transfection reagents that carries two unsaturated chains [35], [97] (Supplemental Fig. S12) even though the lack of the ether-dimethylamine linkage, which could result in a loss of H-bonds, can't be excluded as a cause. Taken together there is growing evidence for a TLR2/TLR1 binding mode of di-C18 lipopolyamines as well as unsaturated lipoglycans and di-acyl lipopeptides that sequesters one lipid chain in each TLR.

### 4.2 Head group-receptor specificity and TLR2 promiscuity

In terms of innate immune response our study shows that the configuration of the head group of lipopolyamines imparts some degree of TLR2 specificity.

The narrow channel at the dimerization interface requires the ligands to remain linear over a distance of at least 6 Å. While aliphatic chains can be accommodated in non-specific ways in hydrophobic pockets, the precise positioning of the head group regulates TLR2 signalling as it can interfere with the dimerization process [82]. Moreover the fact that TLR2 possesses a range of non-lipidic ligands (for instance hyaluronic acid, teichoic acid, and zymosan) also supports the functional importance of the solvent-exposed moiety of the ligand. In other words the interaction of their hydrophilic part is equally critical for TLR2 activation and may differ from lipopeptides. Unfortunately there is no crystal structure to date to describe their respective binding modes.

Recently, some insight was provided by the structure of the TLR2 ectodomain solved in the absence of added ligand [98]. Interestingly the TLR2 hydrophobic pocket was filled with a plasma membrane phospholipid, most likely a phosphatidylcholine (PC). It bound between LRR11 and 12 in the same position as inactive ligands previously characterized [82]. The structure was also solved in the presence of staphylococcal superantigen-like protein 3 (SSL3), a secreted protein that is a potent TLR2 inhibitor. It was shown that SSL3 prevents TLR2 signalling in two ways. The presence of SSL3 prevents ligand binding to TLR2 by partially obstructing the entrance of the lipid-binding pocket. More importantly, the presence of Pam_2_CSK_4_ already bound to TLR2 does not prevent SSL3 binding which in turn prevents the recruitment of TLR6. Using docking analysis, it was then shown that the binding mode of Pam_2_CSK_4_ had to differ significantly from the one observed in the crystal structure of the TLR2/TLR6 complex in order to be compatible with SSL3 binding. This is achieved by the ligand adopting alternative head group interactions with TLR2.

If the ligand itself does not determine its binding mode to TLR2, then this necessarily means that activation is a cooperative mechanism, which includes the TLR2 dimerization partner. In other words TLR2 is not enough to fully engage the ligand. Cooperativity and promiscuity might also be responsible for the heterogeneity of the LPA binding poses predicted here. Further studies will be necessary to confirm that diC18 have indeed such a broad binding spectrum.

## 5 Conclusion

Tested LPAs activate the innate system via TLR2 whether they are delivered in liposome or lipoplex forms. Docking predicts binding poses suitable for either TLR1 or TLR6-driven heterodimerization, while binding energies and biological data seem to favour the former. We determined the structural parameters responsible for TLR2 recognition, extending our results from three very specific lipids to a large number of molecules currently commercialized or proposed to be used as transfection agents. These parameters are most likely to be chosen in the future synthesis of transfection agents because they confer ease of synthesis, while combining biocompatibility and efficiency of transfection. Therefore our study emphasizes the importance of assessing the inflammatory properties of lipoplexes to estimate the safety of transfection, and also aims at warning and guiding on planning new synthesis of non-immunostimulatory nanocarriers. We propose docking analysis as a preliminary screening tool to evaluate pathogen recognition receptor binding for those receptors with available crystal structures. For instance, we predicted that TLR2 activation could no longer be achieved if both LPA chains were unsaturated. Upon biological test, such compounds would provide new non-immunogenic lipoplexes suitable for gene therapy. However, the immunostimulatory properties of saturated LPAs might also be of interest for the development of vaccine adjuvants, especially considering the non-toxicity of LPAs.

## Appendix A Supplementary data

Supplementary figures Image 1
