# Supplementary material for: Toll-like receptor 2 promiscuity is responsible for the immunostimulatory activity of nucleic acid nanocarriers
Source: J Control Release. 2017 Feb 10;247:182–93. doi: 10.1016/j.jconrel.2016.12.029 (PMC5312493; doi:10.1016/j.jconrel.2016.12.029)
Supplement: Supplementary file 1 — Supplementary figures [file mmc1.pdf]

Toll-like receptor 2 promiscuity is responsible for the immunostimulatory activity of nucleic acid nanocarriers

Malvina Pizzuto, Monique Gangloff, Daniel Scherman, Nicholas J. Gay, Virginie Escriou , Jean-Marie Ruysschaert[1]and Caroline Lonez

Supplemental Figures S1-S9. Docking poses of lipopolyamines bound to TLR2. Representations generated by LigPlot+ software.

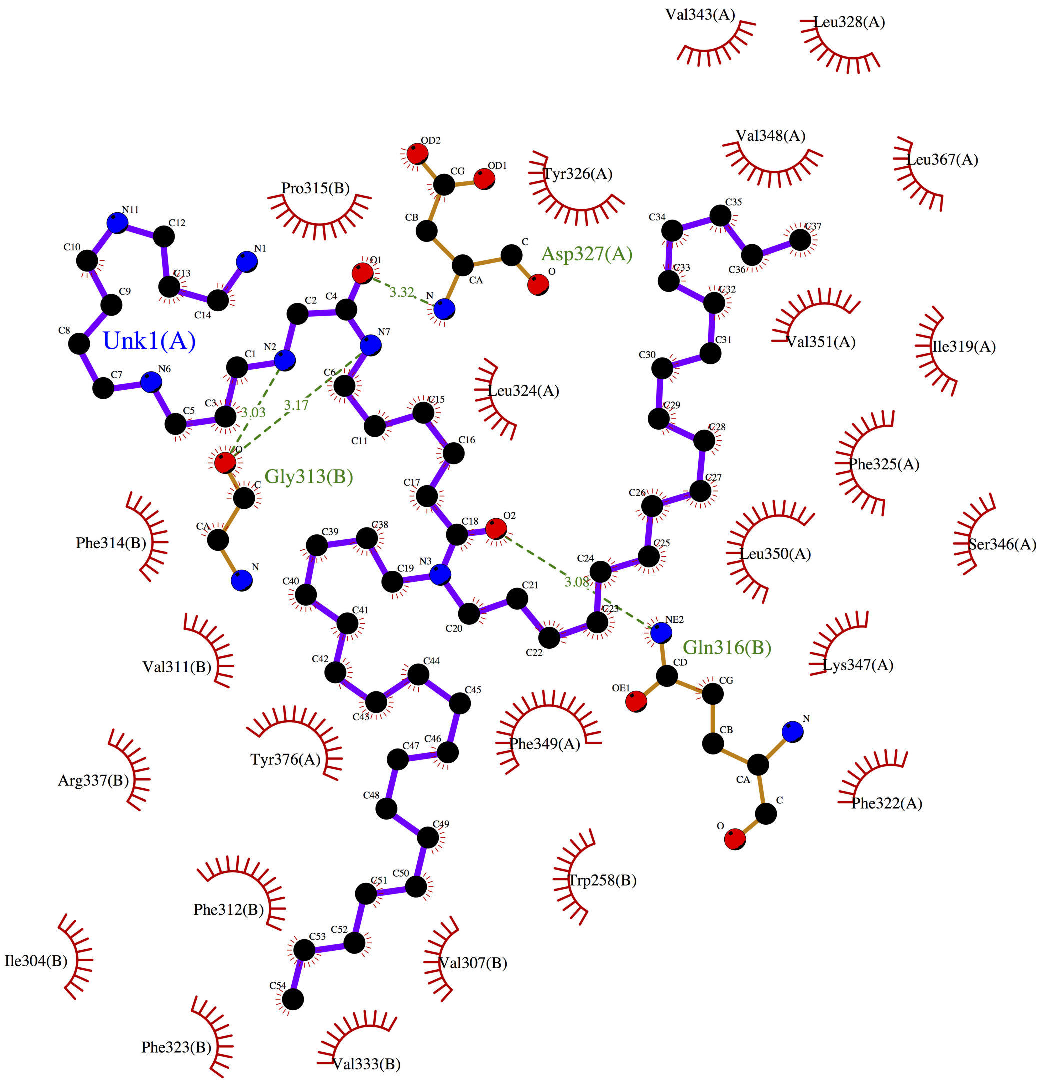

<u>Figure S1.</u> RPR 128506 docking to TLR2/TLR1 with one chains per TLR. The binding energy of this complex is -31.4 kcal/mol.

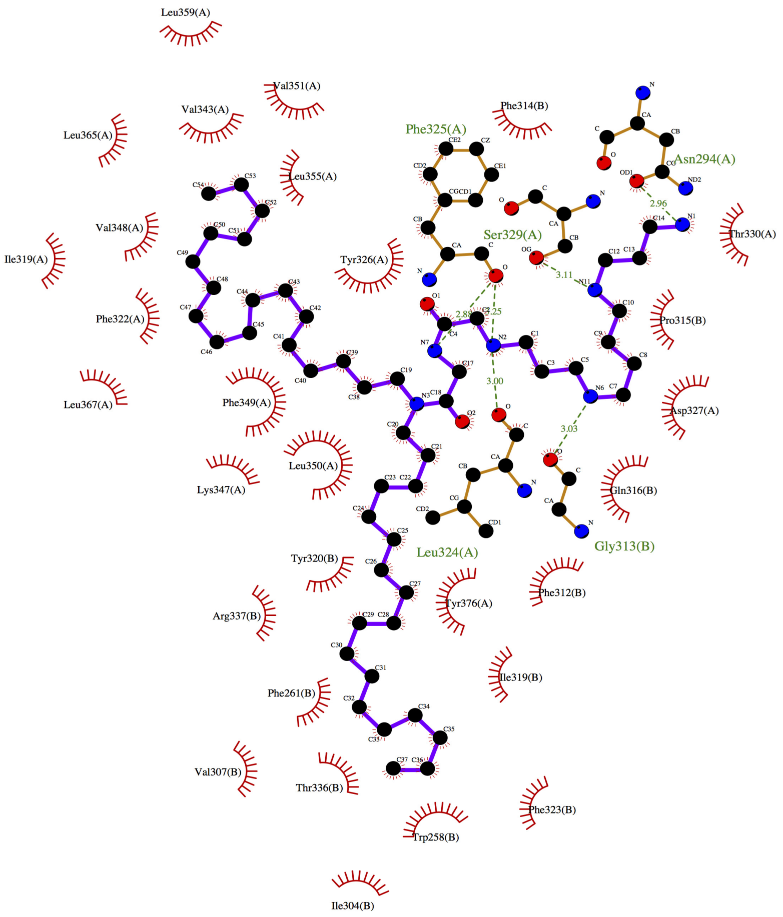

<u>Figure S2</u>. RPR 120535 docking to TLR2/TLR1 with one chain in each TLR. The binding energy of this complex is -32.2 kcal/mol. This is the best pose for this compound. The head group mediates five hydrogen bonds with TLR2 and one with TLR1.

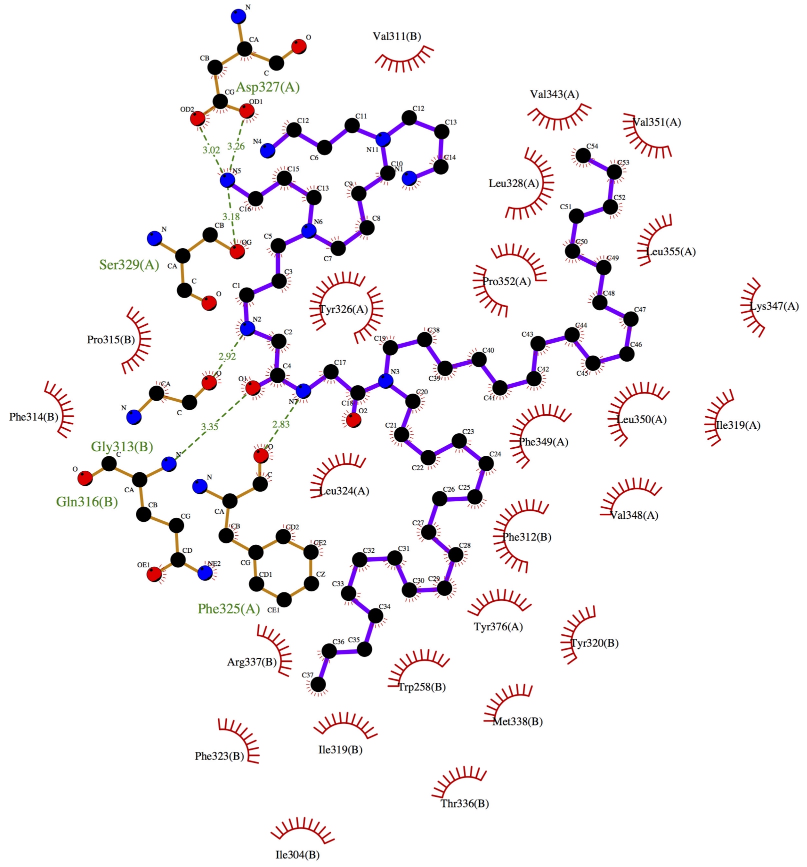

<u>Figure S3.</u> RPR 120525 docking to TLR2/TLR1 with one chain in each TLR. The binding energy of this complex is -32.5 kcal/mol.

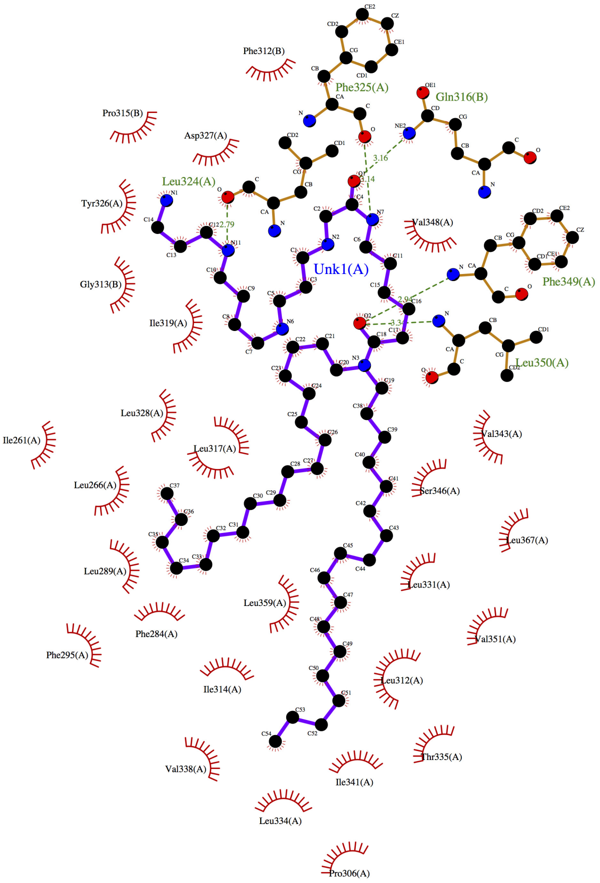

__**Figure S4.**__ **RPR 128506 docking to TLR2/TLR1 with both chains in TLR2.** The binding energy of this complex is -31.9 kcal/mol.

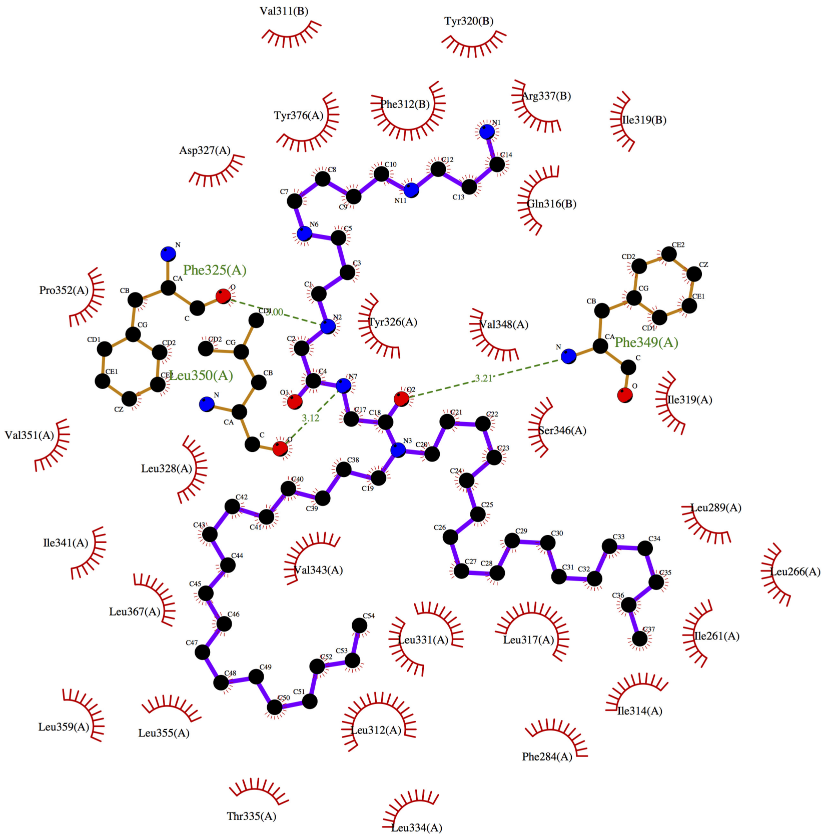

<u>Figure S5.</u> RPR 120535 docking to TLR2/TLR1 with both chains in TLR2. The binding energy of this complex is -31.1 kcal/mol). A hydrogen bond to TLR1 Gln316’ that was found using Chimera is not depicted by LigPlot+.

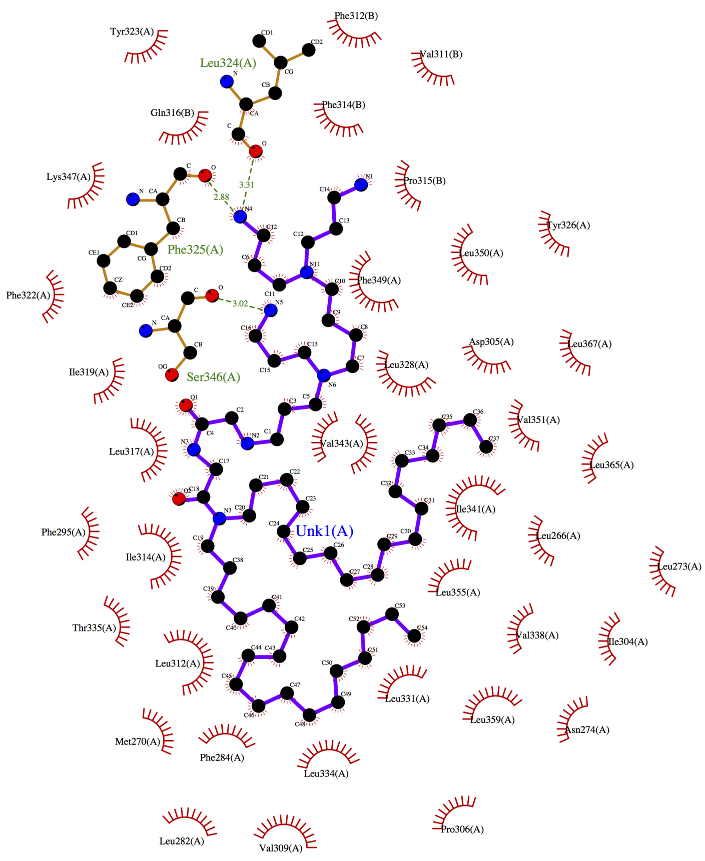

<u>Figure S6.</u> RPR 120525 docking to TLR2/TLR1 with both chains in TLR2. The binding energy of this complex is -32.1 kcal/mol.

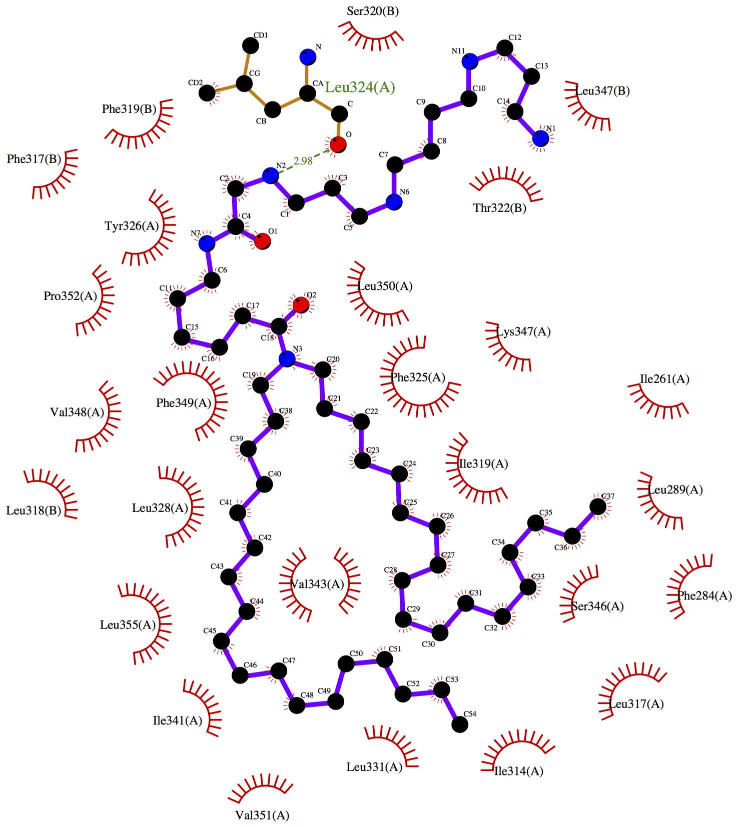

__**Figure S7.**__ **RPR 128506 docking to TLR2/TLR6.** The binding energy of this complex is -29.9 kcal/mol. Additional hydrogen bonds to TLR2 Tyr326 and TLR6 Ser320", Thr322" and Tyr 323" were found using Chimera but is not depicted by LigPlot+.

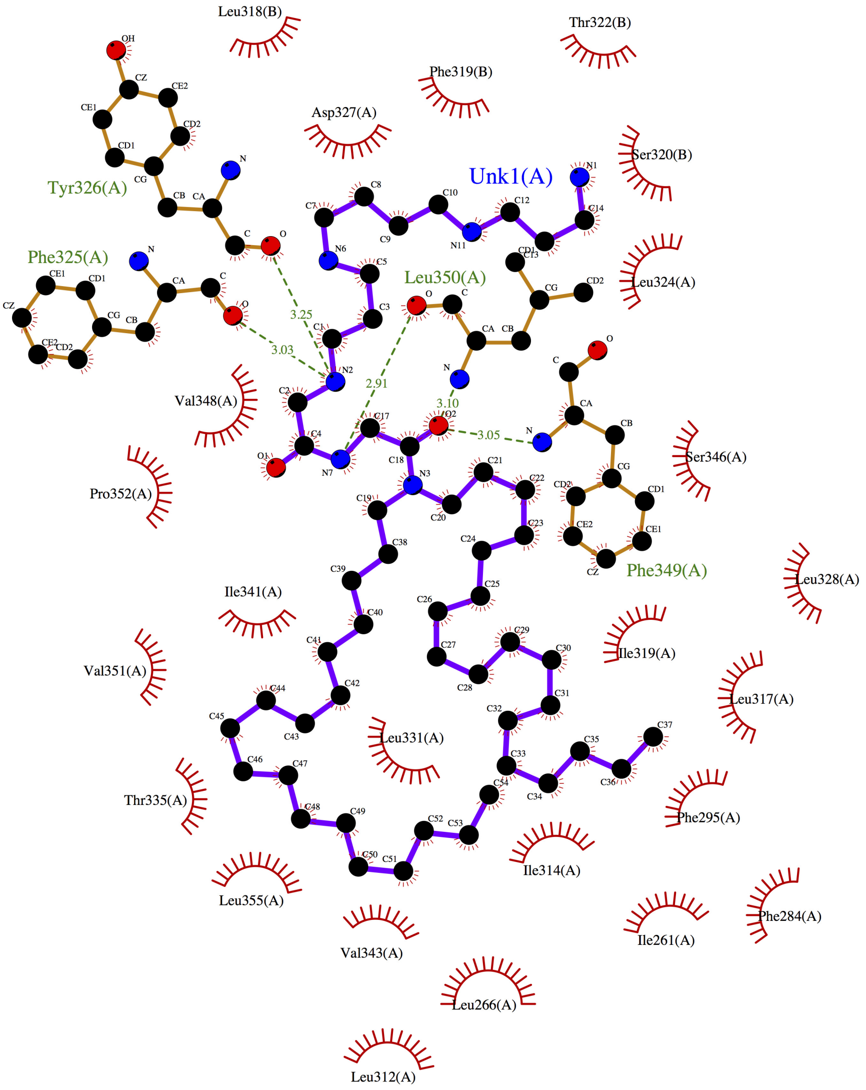

<u>Figure S8.</u> RPR 120535 docking to TLR2/TLR6. The binding energy of this complex is -28.5 kcal/mol. An additional polar contact with TLR6 Ser320 was found using Chimera (right panel) but is not depicted by LigPlot+ (left panel).

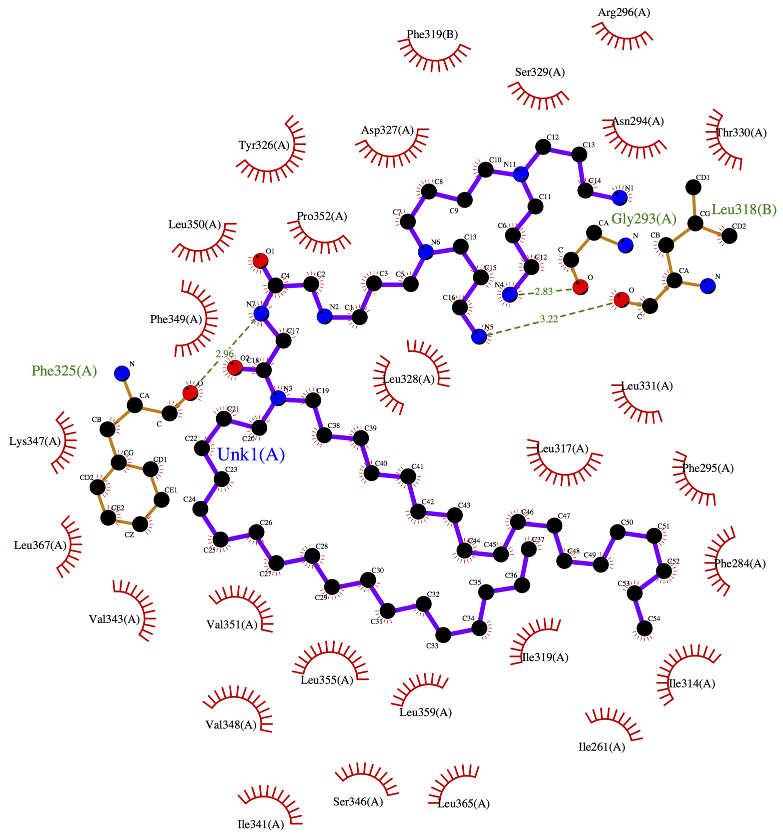

<u>Figure S9.</u> RPR 120525 docking to TLR2/TLR6. The binding energy of this complex is -31.7 kcal/mol.

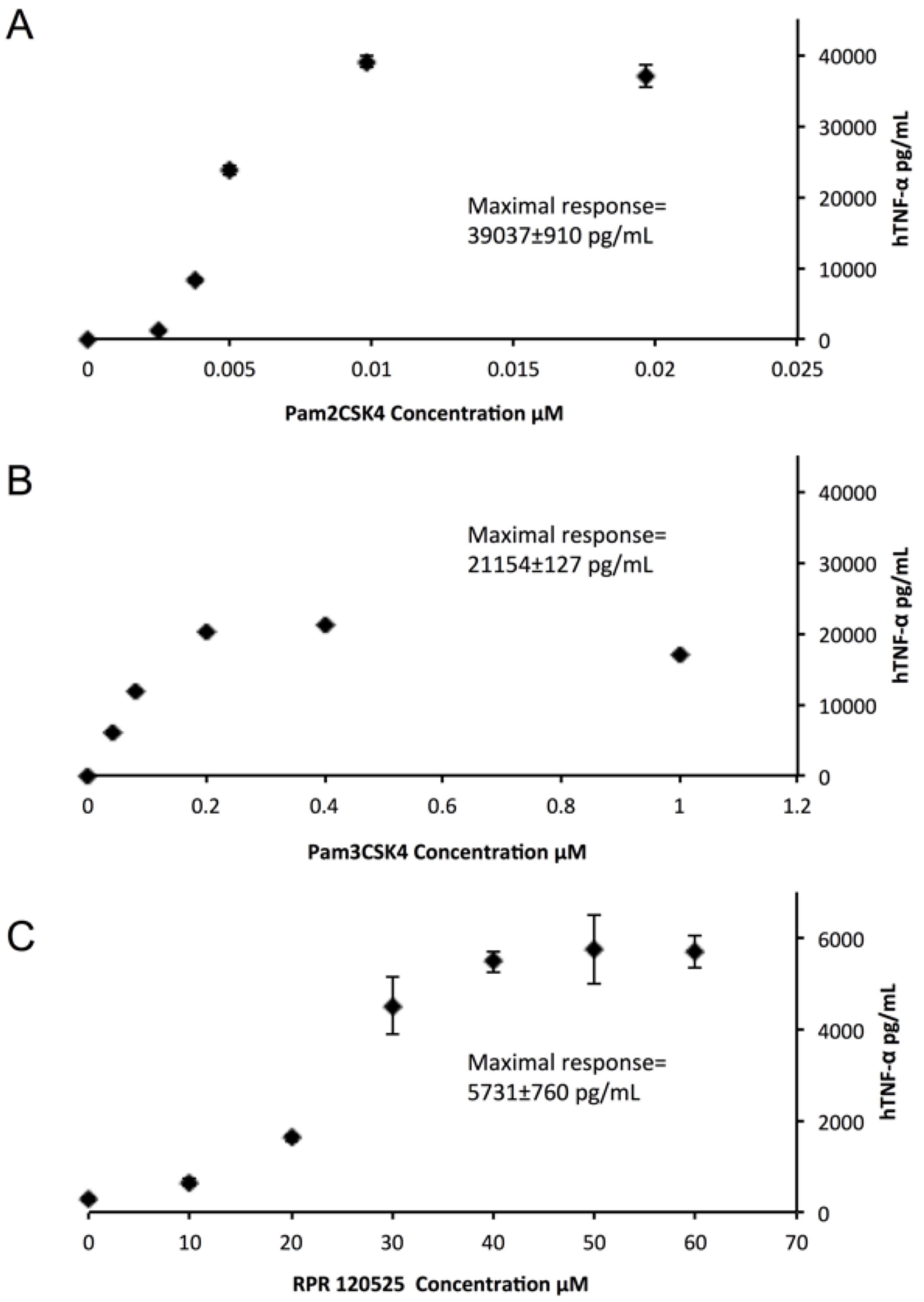

Supplemental Figure S10: RPR 120525 is a weaker agonist then lipopeptides. Primed THP1 cells were incubated for 5h with serum free medium alone (control) or with the indicate amount of (A) Pam_2CSK_4, (B) Pam_3CSK_4 or (C) RPR 120525 cationic lipids in serum free medium, then cell supernatants were collected and TNF-α was quantified using ELISA assay following manufacturer's instructions. Each point represents the mean ± standard deviation of three replicate values (n=3). The experiment is representative of at least 3 replicates.

A

RPR 120535

RPR 208484

B

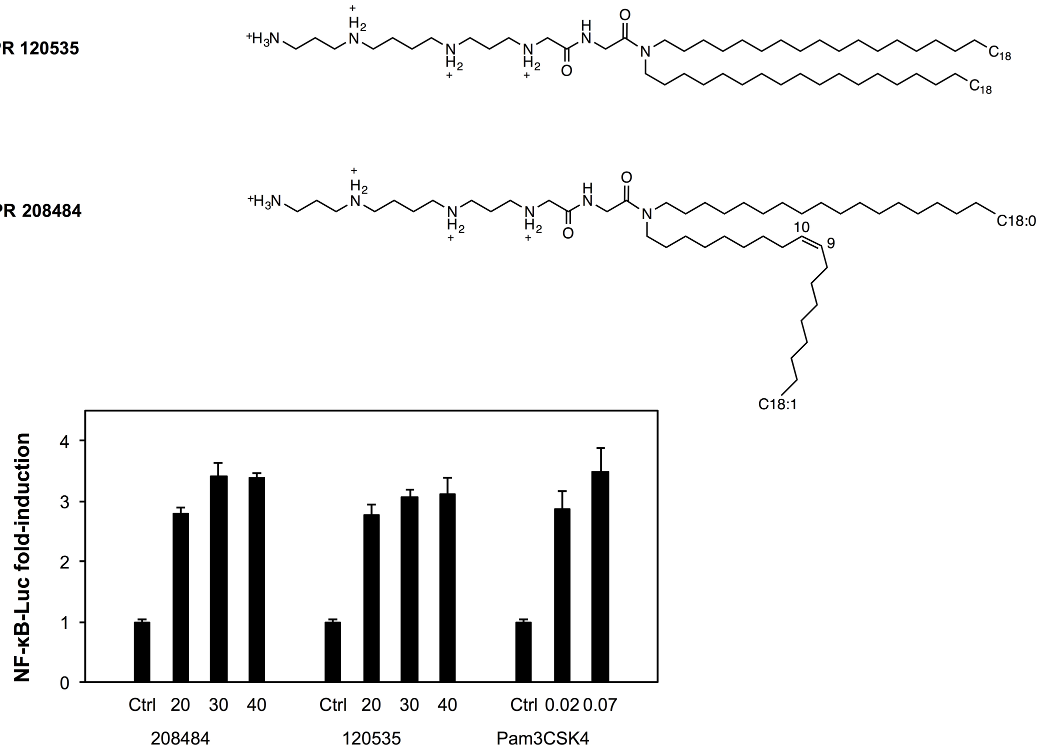

Supplemental Figure S11. RPR 208484 with a single unsaturated alkene chain 18:1 (Δ9-Cis) signals as strongly as its saturated counterpart RPR 120535 and TLR2/TLR1 ligand Pam$_3$CSK$_4$. Raw-blue cells were incubated for 5h with serum free medium alone (C) or with the indicate amount of cationic lipids in µM in serum free medium, then cell supernatants were retired and replaced with complete medium. Supernatants were collected after further 17h of incubation and the NF-κB activation was quantified using Quanti-blue assay following manufacturer's instructions. Each bar represents the mean + standard deviation of three replicate values (n=3). The experiment is representative of at least 3 replicates.

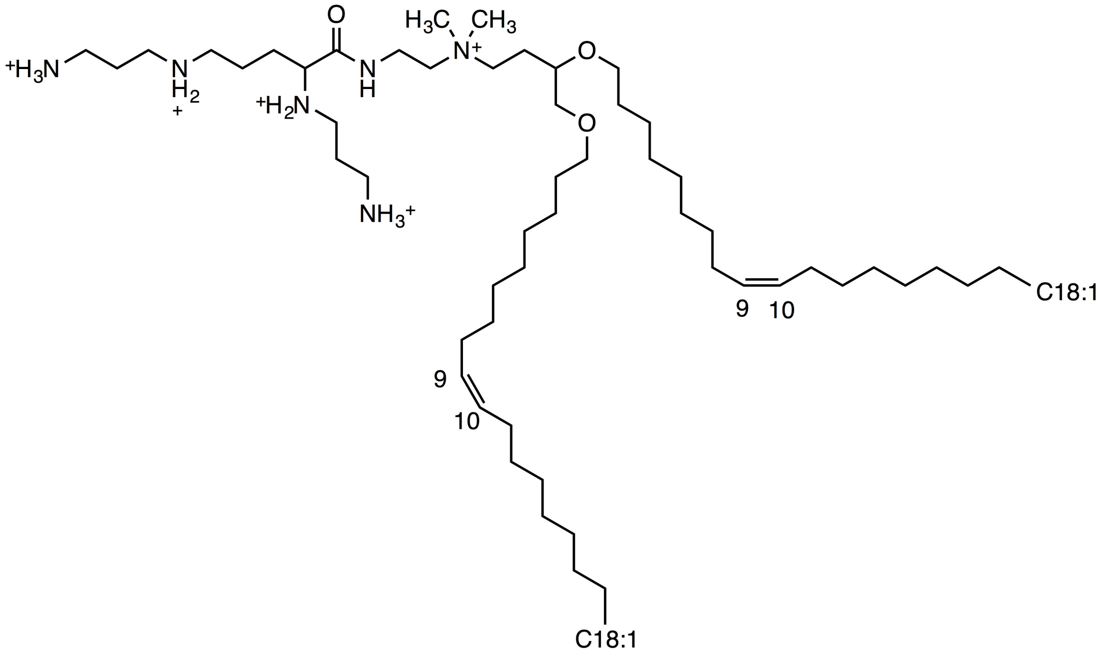

<u>Supplemental Figure S12</u>. Chemical structure of lipofectamine transfection reagent component DOSPA.
